# Paraoxonase 1 concerning dyslipidaemia, cardiovascular diseases, and mortality in haemodialysis patients

**DOI:** 10.1038/s41598-021-86231-0

**Published:** 2021-03-24

**Authors:** Alicja E. Grzegorzewska, Paulina Adamska, Ewa Iwańczyk-Skalska, Kamila Ostromecka, Leszek Niepolski, Wojciech Marcinkowski, Adrianna Mostowska, Wojciech Warchoł, Czesław Żaba, Paweł P. Jagodziński

**Affiliations:** 1 Department of Nephrology, Transplantology and Internal Diseases, Poznan University of Medical Sciences, Przybyszewskiego 49, 60-355 Poznań, Poland; 2 Department of Biochemistry and Molecular Biology, Poznan University of Medical Sciences, 60-781 Poznań, Poland; 3 Nephrology Research Group, Department of Nephrology, Transplantology and Internal Diseases, Poznan University of Medical Sciences, 60-355 Poznań, Poland; 4 B. Braun Avitum Poland, Dialysis Center, 64-300 Nowy Tomyśl, Poland; 5 Fresenius Nephrocare Polska, 60-118 Poznań, Poland; 6 Department of Ophthalmology and Optometry, Poznan University of Medical Sciences, 60-806 Poznań, Poland; 7 Department of Forensic Medicine, Poznan University of Medical Sciences, 60-781 Poznań, Poland

**Keywords:** Genetics, Biomarkers, Cardiology, Diseases, Medical research, Nephrology

## Abstract

Paraoxonase 1 (PON1) is known for preventing atherosclerosis through lipid-modifying features, antioxidant activity, anti-inflammatory, anti-apoptosis, anti-thrombosis, and anti-adhesion properties. Uremic patients requiring haemodialysis (HD) are especially prone to atherosclerosis and its complications. We analysed the PON1 gene (*PON1*) polymorphisms and serum PON1 (paraoxonase) activity concerning dyslipidaemia and related cardiovascular diseases and mortality to show how they associate under uremic conditions modified by maintenance HD treatment. The rs662 AA + AG (OR 1.76, 95%CI 1.10–2.80, P = 0.018), rs854560 TT (OR 1.48, 95%CI 1.04–2.11, P = 0.031), and rs854560 AT + TT (OR 1.28, 95%CI 1.01–1.63, P = 0.040) contributed to the prevalence of atherogenic dyslipidaemia diagnosed by the triglyceride (TG)/HDL-cholesterol ratio ≥ 3.8. The normalized serum PON1 activity positively correlated with atherogenic dyslipidaemia (ẞ 0.67 ± 0.25, P = 0.008). The *PON1* rs854560 allele T was involved in the higher prevalence of ischemic cerebral stroke (OR 1.38, 1.02–1.85, P = 0.034). The *PON1* rs705379 TT genotype contributed to cardiovascular (HR 1.27, 95% CI 1.03–1.57, P = 0.025) and cardiac (HR 1.34, 95% CI 1.05–1.71, P = 0.018) mortality. All P-values were obtained in multiple regression analyses, including clinical variables. Multifaceted associations of *PON1* with dyslipidaemia, ischemic cerebral stroke, and cardiovascular mortality in HD patients provide arguments for the consideration of *PON1* and its protein product as therapeutic targets in the prevention of atherosclerosis and its complications in uremic patients.

## Introduction

Paraoxonase 1 (PON1) is calcium-dependent aryldialkylphosphatase, a 354–355 amino acid glycoprotein, 43–45 kDa^1^. PON1 prevents atherosclerosis through its antioxidant activity, anti-inflammatory action, anti-apoptosis, anti-thrombosis, anti-adhesion, and lipid-modifying properties^2–4^. PON1 stimulates cholesterol efflux, metabolizes oxidized phospholipids in high-density lipoprotein (HDL) and low-density lipoprotein (LDL), prevents lipid-peroxide accumulation on HDL and LDL, and preserves the anti-oxidative HDL function^3,4^. Decreased PON1 activity contributes to elevated plasma levels of homocysteine and homocysteine-thiolactone. The latter can damage proteins by homocysteinylation and involve vascular damage pathology^5,6^.

Uremic patients requiring haemodialysis (HD) are especially prone to atherosclerosis and its complications. Generally, serum PON1 activity^7–10^ and serum PON1 concentrations^11–13^ are decreased in predialysis and dialysis (both HD and peritoneal dialysis) subjects compared to healthy controls. Standardized PON1 activity (the PON1/HDL-cholesterol ratio) may be similar in HD patients and healthy subjects^13^. HD individuals with PON1 concentration below-median showed shorter survival than patients with PON1 concentration equal to or exceeding a median^14^. Therefore, decreased PON1 activity plays an essential role in dyslipidaemia and worse dialysis patients' outcomes. Kidney transplantation can restore PON1 activity, at least partially^8,9,15^, which indicates that factors influencing serum PON1 activity are abolished or ameliorated by recovered renal function.

The human PON1 gene (*PON1*) lies on chromosome 7q21.3^1^. *PON1* promoter region displays several transcription start sites and regulatory elements^16^. The PON1 protein's structural portion is encoded by nine exons that form the primary transcript using typical splice donor and acceptor sites^17^. The *PON1* single nucleotide variant (SNV) designated as rs622 (c.575A>G) is a missense transition resulting in the replacement of glutamine by arginine at amino acid position 192 (p.Gln192Arg). It is well recognized that compared with the unaltered condition, this nucleotide change is associated with increased activities of serum paraoxonase^18^ and chlorpyrifos oxonase^19^, while decreased activities of sarinase and somanase^18^. An impact of *PON1* rs622 polymorphism on arylesterase activity, measured by phenylacetate hydrolysis, was described as approximately the same for AA and GG genotypes^20,21^ or lower for the *PON1* rs622 AA than GG genotype^22^. Another *PON1* missense variant, rs854560 (c.163A>T), leads to leucine-to-methionine substitution at position 55 of the encoded polypeptide (p.Leu55Met)^1^. The altered (c.163 T) *PON1* transcript is less stable than the wild-type transcript, leading to lower amounts of variant mRNA and lower PON1 concentration and activity^23,24^. By contrast, nucleotide variants located in the *PON1* 5′-regulatory region, including the most commonly studied SNVs [rs854572 (− 909G/C), rs705381 (− 162A/G), and rs705379 (− 108C/T)], seem to increase *PON1* expression level and concentration in serum. The − 108 polymorphism is present within a potential factor binding site for Sp1, common in TATA-less genes such as *PON1*^25^.

We aimed to focus on *PON1* rs705379, rs854560, and rs662 SNVs, as well as serum PON1 activity being assessed by catalytic efficiency for paraoxon hydrolysis (paraoxonase activity), concerning dyslipidaemia and atherosclerosis-related cardiovascular complications (coronary heart disease—CHD, type 1 myocardial infarction—MI, ischemic cerebral stroke—ICS) and mortality to show how they associate under uremic conditions modified by maintenance HD treatment. Numerous variables, previously examined as possible influencing *PON1* transcription^16,26–29^ and parameters correlating with serum PON1 concentration or activity^30–34^, were searched in our patients. Additionally, we included in analysis parameters characterizing calcium-phosphate metabolism of HD subjects as human serum PON1 requires the presence of calcium for enzymatic activity^35^, as well as places of settlement that may differ in air pollution with acrolein^36^, a PON1 inactivator^37^. Exploring dyslipidaemia, atherosclerotic diseases, and cardiovascular mortality, possibly positively influenced by favourable *PON1* SNVs and optimal serum PON1 activity, we aimed to comment on *PON1* and PON1 usefulness as future therapeutic targets in the management of HD patients.

## Results

### Patent characteristics

Table 1 presents the patients’ data. In the group tested for serum PON1 activity, there were no individuals taking nandrolone decanoate or hormonal contraceptives. Three subjects supplemented zinc (zinc gluconate, 10 mg/day). All patients were receiving calcium-containing drugs as phosphate-binding medicines. Patients who required erythropoietin-stimulating agent (ESA) medication used epoetin beta.

**Table 1:** Characteristics of HD patients.

Variables tested in both groups	Patients genotyped for *PON1* SNVs (n = 1407)	Patients tested for PON1 activity (n = 93)	P-value^a^	Variables tested in one group	Patients tested for PON1 activity (n = 93)
Clinical data				Clinical and laboratory data	
Male sex (n, %)	782 (55.6%)	55 (59.1%)	0.520	Living in the rural area (n, %)	27 (29.0)
Age (years)	67.0 (11.9–96.1)	66.7 (18.3–86.2)	0.231	LF-HD (n, %)	78 (83.9%)
RRT duration (years)	5.8 (0.01–34.0)	3.9 (0.2–22.3)	0.00003	Mean arterial pressure (mmHg)	113.3 (60–166.7)
Diabetic nephropathy (n, %)	405 (28.8%)	19 (20.4%)	0.096	Cigarette smoking (n, %)	9 (9.7%)
Coronary heart disease (n, %)	542 (38.5% of 1406)	25 (26.9%)	0.027	Urine output (mL/day)	600 (0–2100)
Myocardial infarction (n, %)	299 (21.3% of 1406)	15 (16.1%)	0.292	Treatment with antihypertensive drugs (n, %)	43 (46.2%)
Ischemic cerebral stroke (n, %)	256 (19.2%) of 1335	9 (9.7)	0.026	Treatment with phosphate binding agents (n, %)	79 (84.9%)
Dry body mass (kg), n = 1235	71.5 (31.0–196.0)	81.0 (44.4–144.0)	0.000001	Treatment with vitamin D or vitamin D analogs (n, %)	7 (7.5%)
Body mass index (kg/m^2^)^b^	25.5 (14.3–59.2) n = 1230	26.7 (17.8–51.0)	0.010	Parathyroidectomy (n, %)	1 (1.1%)
Lipid-modifying treatment (n, %) statins (n, %) fibrates (n, %) statins and fibrates (n, %) ezetimibe (n, %) statins + ezetimibe (n, %)	582 (44.3% of 1314) 533 (91.6% of 582) 36 (6.2% of 582) 10 (1.7% of 582) 1 (0.2% of 582) 2 (0.3% of 582)	24 (25.8% of 93) 23 (95.8% of 24) 1 (4.2% of 24) 0 0 0	0.0002 0.712 1.000 1.000 1.000 1.000	Treatment with ESA (n, %)	66 (71.0)
				ESA dose (µg/kg/week)	90 (0–530)
**Type of dyslipidaemia by K/DOQI**				Hemoglobin (g/dL)	11.4 ± 1.3
Hyper-LDL-cholesterolemic (n, %)	388 of 1264 (30.7% )	39 of 93 (41.9%)	0.028	Treatment with cinacalcet (n, %)	6 (6.5%)
Hyper-TG/hyper-non-HDL-cholesterolemic (n, %)	79 of 1264 (6.2% )	5 of 93 (5.4%)	1.000	Vitamin C supplementation (n, %)	2 (2.2%)
Mixed (n, %)	186 of 1264 (14.7% )	14 of 93 (15.1%)	0.545	Zinc supplementation (n, %)	3 (3.2%)
Non-dyslipidemic (n, %)	610 of 1264 (48.3% )	35 of 93 (37.6%)	0.053	Green tea intake (n, %)	4 (4.3%)
**Atherogenic dyslipidaemia (n, %)**	459 of 1264 (36.3% )	39 of 93 (41.9%)	0.316	PON1 activity (U/L)	101.0 (27.7–212.9)
**Laboratory data**				PON1/HDL-cholesterol ratio	2.27 (0.57–7.10)
Total cholesterol (mg/dL)	170.2 (51–626)	183 (83–626)	0.170	Creatinine (mg/dL)	5.7 (1.9–14.4)
HDL-cholesterol (mg/dL)	40 (5–146.8)	44.4 ± 11.7	0.044	Urea (mg/dL)	106 (41–270)
LDL-cholesterol (mg/dL)	95.9 (13.3–512)	104 (35–512)	0.012	C-reactive protein (mg/L)	4.3 (0.3–79.3)
TG (mg/dL)	146.4 (29.8–1363)	157 (65–460)	0.291	Albumin (mg/dL)	4.1 (2.6–4.7)
Non-HDL-cholesterol (mg/dL)	128 (8–593)	135 (41–593)	0.346	Total calcium (mg/dL)	8.8 (6.8–12.5)
LDL/HDL cholesterol ratio	2.38 (0.21–15.52)	2.35 (0.680–15.52)	0.699	Phosphorus (mg/dL)	4.85 (2.32–10.52)
HDL/TC ratio	0.249 (0.045–0.906)	0.250 (0.053–0.530)	0.430	Total ALP (IU/L)	93 (24–321)
TG/HDL-cholesterol ratio	3.60 (0.44–49.71)	3.42 (1.06–17.04)	0.717	Parathyroid hormone (pg/mL)	280 (7.3–1783.8)

*ALP* alkaline phosphatase, *ESA* erythropoietin-stimulating agent, *HD* haemodialysis, *HDL* high-density lipoprotein, *K/DOQI* Kidney Disease Outcomes Quality Initiative, *LDL* low-density lipoprotein, *LF-HD* low-flux haemodialysis, *NA* not available in all patients, *PON1* paraoxonase 1, *PON1* paraoxonase 1 gene, *SNV* single nucleotide variant, *RRT* renal replacement therapy, *TG* triglyceride.

^a^The Kruskal–Wallis and Mann–Whitney U tests were used for comparison of continuous variables. Dichotomous variables were compared using Fisher's exact test.

^b^BMI was not calculated in 5 patients due to amputation of the leg(s).

Individuals of the entire group (n = 1407) and those tested for serum PON1 activity (n = 93) did not differ in the frequency of the *PON1* genotypes (Supplementary Table S1). However, the latter group included subjects being shorter on renal replacement therapy (RRT), having higher body mass, the lower frequency of CHD and ICS, and more rare receiving lipid-modifying medicines (Table 1).

### PON1 SNVs

We obtained polymorphic variants of *PON1* in 1332 patients genotyped for *PON1* rs705379, 1365—for *PON1* rs854560, and 1335—for *PON1* rs662. All SNVs complied with the Hardy–Weinberg equilibrium (HWE). There was a weak linkage disequilibrium (LD, r^2^ < 0.3) between tested SNVs (Supplementary Table S2).

### Probability of obtaining significant associations

Supplementary Table S3 demonstrates the expected sample sizes and odds ratios (ORs) for the likelihood of securing significance at 80% sample power in association analyses between *PON1* SNVs and tested phenotypes. ORs lower than 1.6–1.8 could provide significance at sample power below 80%.

### PON1 SNVs, dyslipidaemia, and related diseases

There were no significant associations between tested *PON1* SNVs and parameters of serum lipid profile. Although bearers of the *PON1* rs662 low activity allele (A) showed lower serum HDL-cholesterol than the rs662 GG homozygotes (Table 2), this relationship did not persist in the multiple regression model including rs662 AA + AG vs. GG (P = 0.106), age (P = 0.721), male gender (P < 0.000001), diabetic nephropathy (P = 0.310), and lipid-modifying treatment (P = 0.005).

**Table 2:** *PON1* SNVs and serum lipid parameters in HD patients.

*PON1* variant	Total cholesterol (mg/dL)	P-value	HDL-cholesterol (mg/dL)	P-value	LDL-cholesterol (mg/dL)	P-value	Triglycerides (mg/dL)	P-value	Non-HDL-cholesterol(mg/dL)	P-value
**rs662**										
AA	168.5 (71.5–626)		40 (5–146.8)		94 (17.4–512)		148 (32–856)		125 (8–593)	
AG	172 (51–368)	0.904^a^	39 (8–103)	0.105^a^	97 (13.3–369)	0.506^a^	144 (35–1363)	0.732^a^	131 (32–329)	0.643^a^
GG	170.1 (84–336)		42 (12–103)		96.8 (33–215)		147.4 (29.8–1105)		127 (42.1–296)	
AA + AG vs. GG	170 (51–626)	0.931^b^	40 (5–146.8)	0.043^b^	95 (13.3–512)	0.660^b^	146.2 (32–1363)	0.678^b^	128 (8–593)	0.508^b^
AA vs. AG + GG	171.5 (51–368)	0.657^b^	40 (8–103)	0.939^b^	97 (13.3–369)	0.383^b^	144 (29.8–1363)	0.440^b^	130.5 (32–329)	0.684^b^
**rs854560**										
AA	169 (65–626)		40.2 (6–118)		94.4 (17–512)		144 (29.8–1105)		127 (40–593)	
AT	170 (51–368)	0.808^a^	40 (7–146.8)	0.193^a^	95.3 (13.3–369)	0.645^a^	144 (32–1363)	0.461^a^	128 (8–329)	0.603^a^
TT	172 (85–337)		40 (5–92)		99.7 (35–239.3)		155 (36.3–856)		129.5 (58–299)	
AT + TT vs. AA	171 (51–368)	0.805^b^	40 (5–146.8)	0.185^b^	96 (13.3–369)	0.950^b^	146.4 (32–1363)	0.583^b^	128 (8–329)	0.625^b^
TT vs. AA + AT	172 (85–337)	0.618^b^	40 (6–146.8)	0.112^b^	95 (13.3–512)	0.364^b^	144 (29.8–1363)	0.218^b^	127.7 (8–593)	0.324^b^
**rs705379**										
CC	170.5 (75–336)		41 (12–103)		97 (20–223)		142 (39–1105)		128 (27–296)	
CT	170 (65–626)	0.608^a^	40 (6–146.8)	0.138^a^	94.6 (17.4–512)	0.536^a^	143 (29.8–1363)	0.275^a^	128 (8–593)	0.973^a^
TT	167.7 (51–363)		39 (5–92)		94 (13.3–369)		157 (32–856)		125.3 (32–313)	
CT + TT vs. CC	169.1 (51–626)	0.635^b^	40 (5–146.8)	0.171^b^	94 (13.3–512)	0.389^b^	146.7 (29.8–1363)	0.197^b^	127 (8–593)	0.927^b^
TT vs. CT + CC	170 (65–626)	0.323^b^	40 (6–146.8)	0.067^b^	95.8 (17.4–512)	0.334^b^	143 (29.8–1363)	0.178^b^	128 (8–593)	0.814^b^

^a^Kruskal-Wallis test for comparison between genotypes.

^b^Mann-Whitney U test for comparison in the specified mode of inheritance.

There were no associations between tested *PON1* SNVs and dyslipidaemic patterns by the Kidney Disease Outcomes Quality Initiative (K/DOQI) criteria (Supplementary Table S4).

The triglyceride (TG)/HDL-cholesterol ratio ≥ 3.8, indicating atherogenic dyslipidaemia^38^, was associated with *PON1* rs662 (the dominant mode) and rs854560 (the additive mode) (Table 3). The association of *PON1* rs662 with atherogenic dyslipidaemia (P = 0.018) persisted in multiple regression analysis including age (P = 0.974), male gender (P = 0.641), diabetic nephropathy (P = 0.327), and lipid-modifying treatment (P = 0.002). In the model comprising the same clinical variables, *PON1* rs854560 TT genotype significantly correlated with atherogenic dyslipidaemia (P = 0.031) together with lipid-modifying treatment (P = 0.015); *PON1* rs854560 AT + TT was also associated with atherogenic dyslipidaemia (P = 0.040) with lipid-modifying treatment (P = 0.016) (Supplementary Table S5). *PON1* rs705379 did not correlate with the frequency of the TG/HDL-cholesterol ratios ≥ 3.8 (Table 3).

**Table 3:** *PON1* polymorphic variants and atherogenic dyslipidaemia diagnosed by the atherogenic index ≥ 3.8.

Genotypes	AI ≥ 3.8 n = 459, 36.3% of all	AI < 3.8 (Reference) n = 805, 63.7% of all	Odds ratio (95% CI), P-value^a^
***PON1 rs662 (575A>G) n = 1195***			
AA vs. AG vs. GG	242 (56%) vs. 164 (38%) vs. 26 (6%)	413 (54.1%) vs. 275 (36%) vs. 75 (9.8%)	0.143^b^
AA + AG vs. GG	406 (94.0%) vs.26 (6.0%)	688 (90.2%) vs.75 (9.8%)	1.702 (1.072, 2.704) 0.023, 60.1%^c^
AA vs. AG + GG	242 (56.0%) vs. 190 (44%)	413 (54.1%) vs. 350 (45.9%)	1.079 (0.851, 1.369), 0.546
***PON1 rs854560 (163A>T) n = 1225***			
TT vs. AT vs. AA	62 (14.1%) vs. 208 (47.3%) vs. 170 (38.6%)	80 (10.2%) vs. 356 (45.4%%) vs. 349 (44.5%)	0.014^b^
AT + TT vs. AA	270 (61.4%) vs. 170 (38.6%)	436 (55.5%) vs. 349 (44.5%)	1.271 (1.002, 1.613) 0.054
TT vs. AA + AT	62 (14.1%) vs. 378 (85.9%)	80 (10.2%) vs. 705 (89.8%)	1.445 (1.014, 2.06) 0.050
***PON1 rs705379 (− 108C>T), n = 1196***			
TT vs. CT vs. CC	98 (23%) vs. 212 (49.6%) vs. 117 (27.4%)	181 (23.5%) vs. 364 (47.3%) vs. 224 (29.1%)	0.792^b^
CT + TT vs. CC	310 (72.6%) vs. 117 (27.4%)	545 (70.9%) vs. 224 (29.1%)	1.089 (0.837, 1.417) 0.548
TT vs. CT + CC	98 (23%) vs. 329 (77%)	181 (23.5%) vs. 588 (76.5%)	0.968 (0.731, 1.281) 0.831

^a^Fisher's exact test.

^b^Cochran-Armitage test.

^c^Sample power for this analysis.

Table 4 shows no significant associations between tested *PON1* SNVs and CHD or type 1 MI. *PON1* rs854560 was significantly associated with ICS (Table 5). In logistic regression, the positive association between the rs854560 T allele and ICS (OR 1.375, 95% CI 1.024–1.847, P = 0.034) was shown together with the annual increase in age (OR 1.025, 95% CI 1.014–1.036, P = 0.000006) and diabetic nephropathy (OR 1.772, 95% CI 1.315–2.388, P = 0.0002). Sex and RRT duration did not yield significance in this model.

**Table 4:** *PON1* polymorphic variants and prevalence of coronary heart disease and type 1 myocardial infarction in HD patients.

Genotypes	CHD	No CHD (Reference)	Odds ratio (95% CI), P-value for no CHD as reference^a^	Myocardial infarction	No myocardial infarction (Reference)	Odds ratio (95% CI), P-value for no myocardial infarctionas reference^a^	Odds ratio (95% CI), P-value for no CHD as reference^a^
***PON1 rs662 (575A>G) n = 1335, P for HWE = 0.05***							
AA vs. AG vs. GG	286 (55.6%) vs. 187 (36.4%) vs. 41 (8%)	444 (54.1%) vs. 308 (37.5%) vs. 69 (8.4%)	0.582^b^	151 (53.7%) vs. 106 (37.7%) vs. 24 (8.5%)	579 (54.9%) vs. 389 (36.9%) vs. 86 (8.2%)	0.715^b^	0.914^b^
AA + AG vs. GG	473 (92%) vs. 41 (8.0%)	752 (91.6%) vs. 69 (8.4%)	1.059 (0.707, 1.584) 0.838	257 (91.5%) vs.24 (8.5%)	968 (91.8%) vs. 86 (8.2%)	0.951 (0.593, 1.527) 0.808	0.983 (0.605, 1.597) 1.000
AA vs. AG + GG	286 (55.6%) vs. 228 (44.4%)	444 (54.1%) vs.377 (45.9%)	1.065 (0.853, 1.329) 0.611	151 (53.7%) vs.130 (46.3%)	579 (54.9%) vs. 475 (45.1%)	0.953 (0.732, 1.241) 0.736	0.986 (0.752, 1.294) 0.945
***PON1 rs854560 (163A>T) n = 1365, P for HWE = 0.12***							
TT vs. AT vs. AA	60 (11.4%) vs. 246 (46.6%) vs. 222 (42%)	99 (11.8%) vs. 377 (45%) vs. 361 (43.1%)	0.867^b^	37 (12.7%) vs. 120 (41.2%) vs. 134 (46%)	122 (11.4%) vs. 503 (46.8%) vs. 449 (41.8%)	0.514^b^	0.659^b^
AT + TT vs. AA	306 (58%) vs. 222 (42%)	476 (56.9%) vs. 361 (43.1%)	1.045 (0.839, 1.303) 0.736	157 (54%) vs. 134 (46%)	625 (58.2%) vs. 449 (41.8%)	0.842 (0.649, 1.092) 0.205	0.990 (0.802, 1.223) 0.957
TT vs. AA + AT	60 (11.4%) vs. 468 (88.6%)	99 (11.8%) vs. 738 (88.2%)	0.956 (0.68, 1.344) 0.862	37 (12.7%) vs. 254 (87.3%)	122 (11.4%) vs. 952 (88.6%)	1.137 (0.767, 1.684) 0.537	1.000 (0.72, 1.389) 1.000
***PON1 rs705379 (− 108C>T), n = 1332, P for HWE = 0.25***							
TT vs. CT vs. CC	126 (24.7%) vs. 235 (46.1%) vs. 149 (29.2%)	182 (22.1%) vs. 408 (49.6%) vs. 232 (28.2%)	0.151^b^	74 (26.6%) vs. 115 (41.4%) vs. 89 (32%)	234 (22.2%) vs. 528 (50.1%) vs. 292 (27.7%)	0.982^b^	0.891 ^b^
CT + TT vs. CC	361 (70.8%) vs. 149 (29.2%)	590 (71.8%) vs. 232 (28.2%)	0.953 (0.746, 1.216) 0.708	189 (68.0%) vs. 89 (32.0%)	762 (72.3%) vs. 292 (27.7%)	0.814 (0.611, 1.083) 0.157	0.928 (0.735, 1.173) 0.049
TT vs. CT + CC	126 (24.7%) vs. 384 (75.3%)	182 (22.1%) vs. 640 (77.9%)	1.154 (0.89, 1.496) 0.285	74 (26.6%) vs. 204 (73.4%)	234 (22.2%) vs. 820 (77.8%)	1.271 (0.939, 1.721) 0.129	1.150 (0.897, 1.474) 0.276

*CHD* coronary heart disease, *HD* haemodialysis, *PON1*—paraoxonase 1 gene.

^a^Fisher's exact test.

^b^Cochran-Armitage test.

**Table 5:** *PON1* polymorphic variants and the cerebral stroke prevalence in HD patients.

Genotypes	Cerebral stroke	No cerebral stroke (Reference)	Odds ratio (95% CI), P-value^a^
***PON1 rs662 (575A > G)***			
AA vs. AG vs. GG	139 (57.4%) vs. 90 (37.2%) vs. 13 (5.4%)	556 (54.2%) vs. 381 (37.2%) vs. 88 (8.6%)	0.161^b^
AA + AG vs. GG	229 (94.6%) vs. 13 (5.4%)	937 (91.4%) vs. 88 (8.6%)	1.654 (0.908, 3.015) 0.113
AA vs. AG + GG	139 (57.4%) vs 103 (42.6%)	556 (54.2%) vs. 469 (45.8%)	1.138 (0.858, 1.511) 0.390
***PON1 rs854560 (163A>T)***			
TT vs. AT vs. AA	38 (15.4%) vs. 123 (49.8%) vs. 86 (34.8%)	118 (11.3%) vs. 473 (45.2%) vs. 456 (43.6%)	0.005^b^
AT + TT vs. AA	161 (65.2%) vs. 86 (34.8%)	591 (56.4%) vs. 456 (43.6%)	1.444 (1.082, 1.928) 0.012, 69.9%^c^
TT vs. AA + AT	38 (15.4%) vs. 209 (84.6%)	118 (11.3%) vs. 929 (88.7%)	1.431 (0.964, 2.125) 0.082
***PON1 rs705379 (− 108C>T)***			
TT vs. CT vs. CC	63 (26.4%) vs. 106 (44.4%) vs. 70 (29.3%)	239 (23.3%) vs. 503 (49%) vs. 284 (27.7%)	0.778^b^
CT + TT vs. CC	169 (70.7%) vs. 70 (29.3%)	742 (72.3%) vs. 284 (27.7%)	0.924 (0.678, 1.260) 0.632
TT vs. CT + CC	63 (26.4%) vs. 176 (73.6%)	239 (23.3%) vs. 787 (76.7%)	1.179 (0.854, 1.627) 0.313

*HD* haemodialysis, *PON1* paraoxonase 1 gene.

^a^Fisher's exact test.

^b^Cochran-Armitage test.

^c^Sample power for this analysis.

### Serum PON1 and patient data

In unadjusted analyses, the lower serum PON1 activity, the higher frequency of mixed dyslipidaemia by K/DOQI guidelines^39^, and the higher serum TG levels (Table 6). The PON1/HDL-cholesterol ratio correlated positively with male sex, cigarette smoking, presence of atherogenic dyslipidaemia, and values of the TG/HDL-cholesterol ratio (Table 7).

**Table 6:** Correlates of serum PON1 activity (U/L) in HD patients (n = 93).

Parameter	Unadjusted percent change in PON1activity		Adjusted percent change in PON1activity^a^	
	β^b^ ± SE	P-value	β ± SE	P-value
**Clinical data**				
Male sex	6.6 ± 7.4	0.375	4.7 ± 7.4	0.527
Age (per 10 years)	1.7 ± 2.5	0.490	− 0.99 ± 2.93	0.737
Living in the rural area	10.0 ± 7.8	0.215	9.6 ± 8	0.233
RRT duration (per 1 year)	0.2 ± 0.8	0.829	− 0.29 ± 0.89	0.743
LF-HD	− 7.9 ± 9.9	0.428	− 5 ± 10.1	0.623
Diabetic nephropathy	4.4 ± 9.0	0.628	1.5 ± 8.9	0.869
Coronary heart disease	− 0.2 ± 8.2	0.983	− 4.7 ± 8.2	0.572
Myocardial infarction	− 1.7 ± 9.9	0.866	− 1.8 ± 9.9	0.856
Ischemic cerebral stroke	20.5 ± 12.2	0.096	16 ± 12.4	0.201
Mean arterial pressure (per 10 mmHg)	0.72 ± 2.89	0.804	1.7 ± 3.0	0.571
Dry body mass (per 10 kg)	− 1.0 ± 2.3	0.659	− 1.6 ± 2.5	0.510
Body mass index (per 5 kg/m^2^)	0.2 ± 3.9	0.963	− 0.7 ± 4.0	0.851
Cigarette smoking	21.1 ± 12.2	0.086	24.4 ± 12.4	0.052
Urine output (per 300 mL/day)	− 1.2 ± 2.1	0.552	− 2.0 ± 2.0	0.339
Lipid-modifying treatment	7.5 ± 8.3	0.371	6.0 ± 8.2	0.467
- treatment with statins	7.3 ± 8.4	0.389	6.6 ± 8.3	0.429
Treatment with antihypertensive drugs	8.0 ± 7.3	0.276	12.5 ± 7.3	0.093
Treatment with phosphate binding agents	4.7 ± 10.2	0.648	6.2 ± 10.1	0.540
Treatment with calcium-based phosphate binders	4.7 ± 10.2	0.648	6.2 ± 10.1	0.540
Treatment with vitamin D (alfacalcidol–2 cases) or vitamin D analogs (paricalcitol—5 cases)	− 5.0 ± 13.8	0.716	− 7.9 ± 14.3	0.583
Treatment with ESA	5.0 ± 8.0	0.532	6.7 ± 7.9	0.401
ESA dose (per 1 µg/kg/week)	0.036 ± 0.029	0.217	0.041 ± 0.028	0.150
Treatment with cinacalcet	− 5.3 ± 14.9	0.721	0.1 ± 14.7	0.995
Vitamin C supplementation	7.6 ± 25.2	0.763	6.3 ± 24.6	0.799
Zinc supplementation	27.1 ± 20.5	0.189	29.8 ± 20.7	0.155
Green tea intake	− 0.1 ± 18.0	0.997	3.2 ± 17.9	0.858
**Type of dyslipidaemia by K/DOQI**				
Hyper-LDL-cholesterolemic	7.25 ± 7.36	0.328	5.4 ± 7.3	0.467
Hyper-TG/hyper-non-HDL-cholesterolemic	6.48 ± 16.18	0.690	9.3 ± 16.6	0.577
Mixed	− 21.4 ± 10.0	0.035	− 16.4 ± 10.1	0.109
Non-dyslipidemic	2.72 ± 7.53	0.719	1.3 ± 7.5	0.861
Atherogenic dyslipidaemia	− 5.25 ± 7.38	0.479	− 7.5 ± 7.5	0.323
**Laboratory data**				
Total cholesterol (per 10 mg/dL)	− 1.0 ± 0.6	0.066	− 0.77 ± 0.56	0.170
HDL cholesterol (per 10 mg/dL)	− 3.9 ± 3.1	0.218	− 1.7 ± 3.4	0.610
LDL cholesterol (per 10 mg/dL)	0.31 ± 0.63	0.625	− 0.20 ± 0.62	0.752
TG (per 10 mg/dL)	− 1.06 ± 0.49	0.034	− 0.94 ± 0.48	0.056
Non-HDL-cholesterol (per 10 mg/dL)	− 0.91 ± 0.56	0.108	− 0.71 ± 0.55	0.204
LDL/HDL cholesterol (per 1.0)	0.79 ± 1.79	0.658	0.67 ± 1.81	0.712
HDL/TC (per 5%)	41.0 ± 42.1	0.332	46.4 ± 42	0.272
TG/HDL-cholesterol (per 1.0)	− 1.5 ± 1.4	0.288	− 1.8 ± 1.4	0.206
Creatinine (per 1 mg/dL)	− 0.66 ± 1.57	0.674	− 1.2 ± 2.0	0.551
Urea (per 10 mg/dL)	− 0.35 ± 0.99	0.723	0.53 ± 1.10	0.628
C-reactive protein (per 1 mg/L)	− 0.25 ± 0.28	0.370	− 0.40 ± 0.28	0.150
Albumin (per 1 g/dL)	5.3 ± 10.4	0.609	3.1 ± 10.4	0.767
Total calcium (per 1 mg/dL)	− 1.2 ± 4.1	0.774	− 1.1 ± 4.1	0.783
Phosphorus (per 1 mg/dL)	− 3.9 ± 2.0	0.060	− 4.0 ± 2.0	0.054
Total ALP (per 100 IU/L)	6.6 ± 6.3	0.295	5.5 ± 6.5	0.394
Parathyroid hormone (per 1000 pg/mL)	2.0 ± 8.2	0.807	1.9 ± 8.6	0.830
Haemoglobin (per 1 g/dL)	1.7 ± 2.9	0.546	1.4 ± 2.8	0.628

*ALP* alkaline phosphatase, *ESA* erythropoietin-stimulating agent, *HD* haemodialysis, *HDL* high-density lipoprotein, *LDL* low density lipoprotein, *LF-HD* low-flux haemodialysis, *PON1* paraoxonase 1 gene, *RRT* renal replacement therapy, *TG* triglycerides.

^a^Gender, cigarette smoking, urine output, living in a rural area, and serum phosphorus were used for adjustment in the multiple regression analysis.

^b^β coefficient can be interpreted as the percent change expected in serum PON1activity for each unit change in the tested variable.

**Table 7:** Correlates of the normalized serum PON1 activity (PON1/HDL-cholesterol ratio).

Parameter	Unadjusted percent change in PON1/HDL-cholesterol ratio		Adjusted percent change in PON1/HDL-cholesterol ratio	
	β ^b^ ± SE	P value	β ± SE	P value
**Clinical data**				
Male sex	0.56 ± 0.25	0.029	0.51 ± 0.24	0.037
Age (per 10 years)	0.050 ± 0.087	0.598	− 0.006 ± 0.097	0.955
Living in the rural area (n, %)	0.53 ± 0.27	0.056	0.55 ± 0.26	0.039
RRT duration (per 1 year)	0.031 ± 0.027	0.264	0 ± 0.029	0.999
LF-HD	− 0.38 ± 0.34	0.268	− 0.19 ± 0.33	0.577
Diabetic nephropathy	0.43 ± 0.31	0.170	0.33 ± 0.29	0.258
Coronary heart disease	0.26 ± 0.28	0.363	0.06 ± 0.27	0.828
Myocardial infarction	− 0.43 ± 0.34	0.212	− 0.4 ± 0.32	0.216
Ischemic cerebral stroke	0.21 ± 0.43	0.618	− 0.08 ± 0.41	0.843
Mean arterial pressure (per 10 mmHg)	− 0.035 ± 0.100	0.730	− 0.001 ± 0.101	0.989
Dry body mass (per 10 kg)	0.065 ± 0.081	0.423	0.031 ± 0.082	0.706
Body mass index (per 5 kg/m^2^)	0.13 ± 0.14	0.346	0.088 ± 0.131	0.501
Cigarette smoking	0.86 ± 0.42	0.043	0.93 ± 0.41	0.024
Urine output (per 300 mL/day)	− 0.12 ± 0.07	0.098	− 0.14 ± 0.07	0.046
Lipid-modifying treatment - treatment with statins	− 0.005 ± 0.288 0.027 ± 0.292	0.987 0.928	− 0.04 ± 0.27 0.024 ± 0.276	0.878 0.931
Treatment with antihypertensive drugs	0.11 ± 0.25	0.659	0.27 ± 0.24	0.280
Treatment with phosphate binding agents	0.04 ± 0.35	0.905	0.05 ± 0.34	0.876
Treatment with vitamin D (alfacalcidol—2 cases) or vitamin D analogues (paricalcitol—5 cases)	− 0.42 ± 0.48	0.383	− 0.71 ± 0.47	0.129
Treatment with ESA	− 0.04 ± 0.28	0.878	0.05 ± 0.26	0.860
ESA dose (per 1 µg/kg/week)	− 0.0004 ± 0.0010	0.695	− 0.0001 ± 0.0010	0.894
Treatment with cinacalcet	− 0.65 ± 0.51	0.203	− 0.40 ± 0.48	0.409
Vitamin C supplementation	− 0.61 ± 0.87	0.483	− 0.67 ± 0.81	0.408
Zinc supplementation	1.18 ± 0.70	0.098	1.5 ± 0.67	0.029
Green tea intake	0.04 ± 0.62	0.943	0.11 ± 0.59	0.855
**Type of dyslipidaemia by K/DOQI**				
Hyper-LDL-cholesterolemic	0.08 ± 0.26	0.741	0.04 ± 0.24	0.882
Hyper-TG/hyper-non-HDL-cholesterolemic	− 0.05 ± 0.56	0.927	− 0.29 ± 0.55	0.604
Mixed	0.28 ± 0.35	0.430	− 0.1 ± 0.34	0.779
Non-dyslipidemic	0.08 ± 0.26	0.773	0.07 ± 0.25	0.774
**Atherogenic dyslipidaemia** TG/HDL-cholesterol (per 1.0)	0.67 ± 0.25 0.12 ± 0.05	0.008 0.018	0.54 ± 0.24 0.093 ± 0.047	0.028 0.049
**Laboratory data**^**c**^				
Total cholesterol (per 10 mg/dL)	− 0.033 ± 0.019	0.091	− 0.019 ± 0.019	0.298
LDL cholesterol (per 10 mg/dL)	0.014 ± 0.022	0.525	0.016 ± 0.021	0.432
TG (per 10 mg/dL)	− 0.012 ± 0.017	0.500	− 0.009 ± 0.016	0.591
Non-HDL-cholesterol (per 10 mg/dL)	0.011 ± 0.020	0.560	− 0.003 ± 0.018	0.887
Creatinine (per 1 mg/dL)	0.056 ± 0.051	0.279	0.000 ± 0.061	1.000
Urea (per 10 mg/dL)	− 0.001 ± 0.034	0.976	0.005 ± 0.036	0.894
C-reactive protein (per 1 mg/L)	− 0.0065 ± 0.0097	0.502	− 0.011 ± 0.009	0.222
Albumin (per 1 mg/dL)	0.31 ± 0.36	0.390	0.08 ± 0.34	0.807
Total calcium (per 1 mg/dL)	0.03 ± 0.14	0.839	− 0.02 ± 0.14	0.901
Phosphorus (per 1 mg/dL)	− 0.078 ± 0.071	0.275	− 0.083 ± 0.068	0.225
Total ALP (per 100 IU/L)	0.25 ± 0.23	0.267	0.18 ± 0.22	0.411
Parathyroid hormone (per 1000 pg/mL)	0.21 ± 0.28	0.465	0.17 ± 0.28	0.541
Hemoglobin (per 1 g/dL)	0.079 ± 0.099	0.424	0.057 ± 0.093	0.541

*ALP* alkaline phosphatase, *ESA* erythropoietin-stimulating agent, *HD* haemodialysis, *HDL* high-density lipoprotein, LDL—low-density lipoprotein, *LF-HD* low-flux haemodialysis, *PON1* paraoxonase 1 gene, *RRT* renal replacement therapy, *TG* triglycerides.

^a^Gender, cigarette smoking, urine output, living in a rural area, and serum phosphorus were used for adjustment in the multiple regression analysis.

^b^β coefficient can be interpreted as the percent change expected in serum PON1activity for each unit change in the tested variable.

^c^HDL-cholesterol and lipid indices calculated using HDL-cholesterol were not included in this analysis due to mathematical coupling.

After adjustment for gender, cigarette smoking, urine output, living in a rural area, and serum phosphorus, significance persisted between normalized serum PON1activity and male sex, cigarette smoking*,* and atherogenic dyslipidaemia (and the TG/HDL-cholesterol ratio)*,* as well as appeared for living in rural areas, urine output, and zinc supplementation.

### Survival analyses

Eight hundred four patients died during their RRT period lasting 0.01–34.0 years (Table 1). Cardiovascular mortality was a cause of death in 485 patients (354 subjects died from cardiac reasons, 131—from vascular diseases).

**Figure 1:**
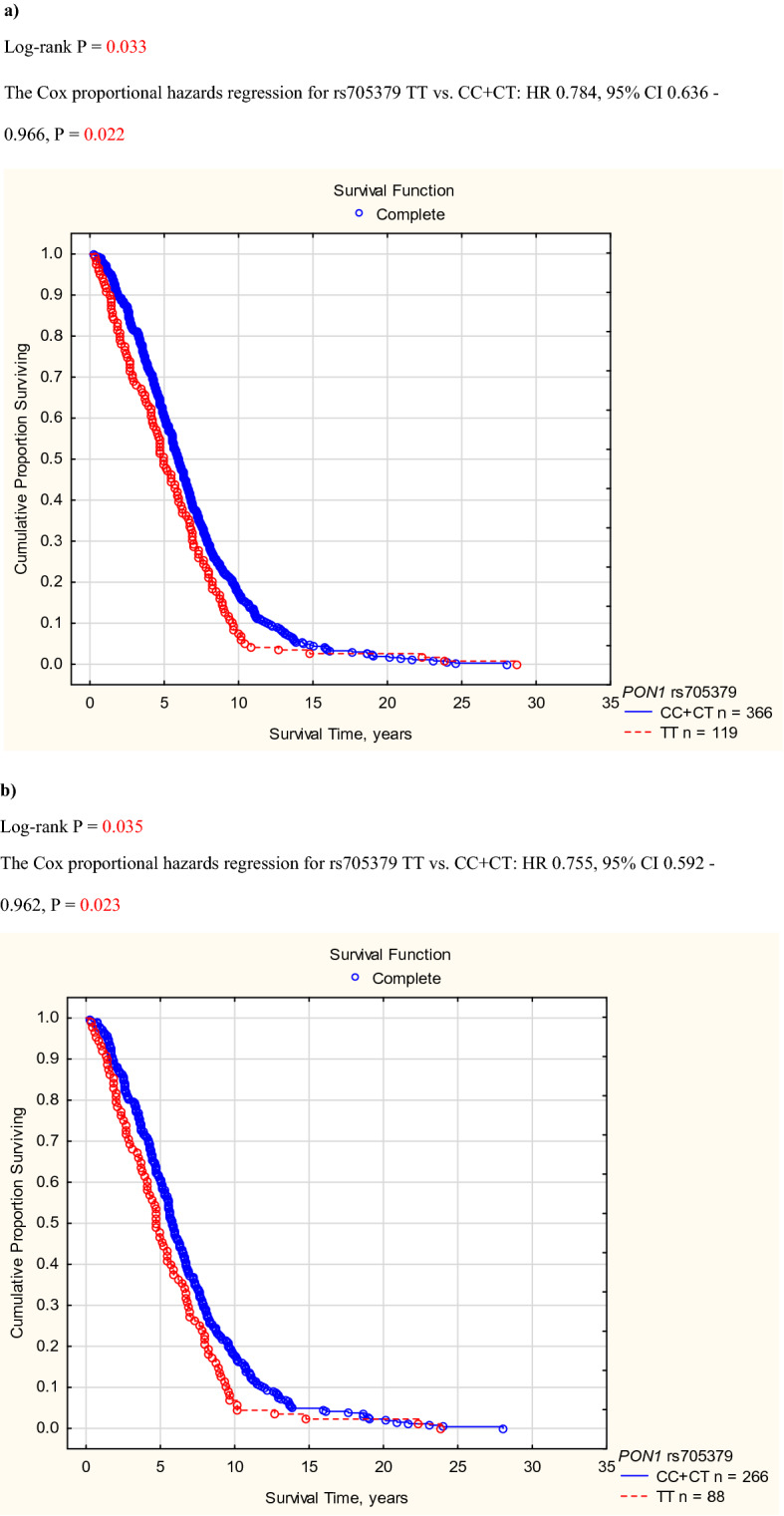

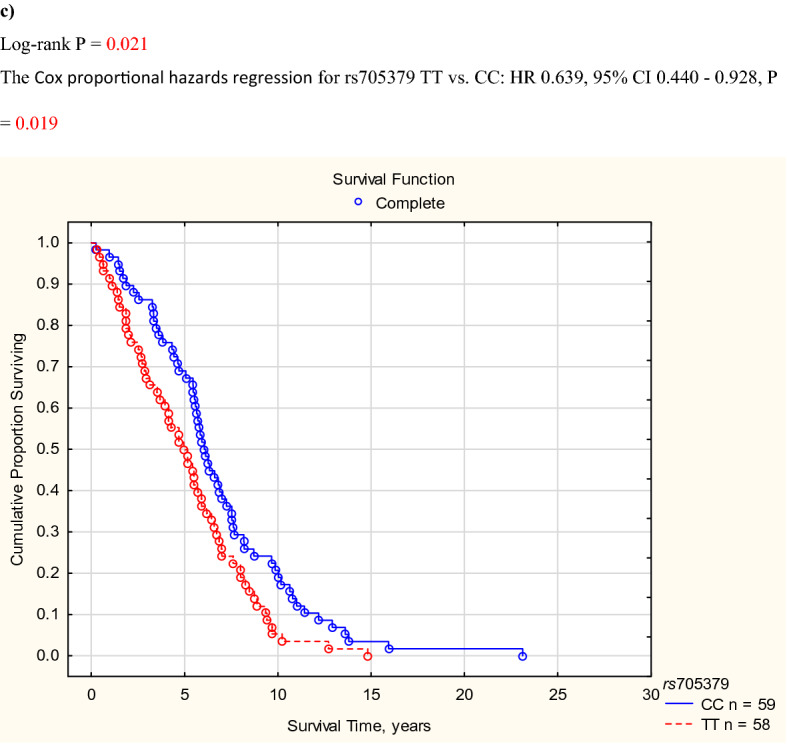
*PON1* rs705379 and cumulative proportion surviving for cardiovascular diseases in HD patients. (**a**) All cardiovascular diseases. (**b**) Cardiac diseases. (**c**) Coronary heart disease and its complications. (**a)** Log-rank P = 0.033. The Cox proportional hazards regression for rs705379 TT vs. CC + CT: HR 0.784, 95% CI 0.636–0.966, P = 0.022. (**b)** Log-rank P = 0.035. The Cox proportional hazards regression for rs705379 TT vs. CC + CT: HR 0.755, 95% CI 0.592–0.962, P = 0.023. (**c)** Log-rank P = 0.021. The Cox proportional hazards regression for rs705379 TT vs. CC: HR 0.639, 95% CI 0.440—0.928, P = 0.019.

If cardiovascular survival probability was compared between genotypes of *PON1* rs705379 (TT vs. CT vs. CC), the log-rank test revealed a significant difference (P = 0.025). Homozygotes TT of *PON1* rs705379 revealed lower cardiovascular survival than the C allele bearers (Fig. 1a). A significance was shown for cardiac deaths in the recessive mode of inheritance (Fig. 1b) and mortality from CHD and its complications between the TT and CC genotypes (Fig. 1c) but not for vascular deaths, also if deaths from ICS were analysed separately (n = 98). These significances yielded P < 0.05 in the Cox proportional hazards models including age, gender, diabetic nephropathy, and rs705379 TT vs. CC + CT (HR 1.269, 95% CI 1.030–1.565, P = 0.025 for cardiovascular mortality; HR 1.343, 95% CI 1.053–1.713, P = 0.018 for cardiac mortality). Significantly associated with cardiovascular and cardiac mortalities were also age (HR 1.013, 95% CI 1.006–1.021, P = 0.006 for cardiovascular; HR 1.014, 95% CI 1.004–1.023, P = 0.004 for cardiac mortality) and diabetic nephropathy (HR 1.366, 95% CI 1.134–1.645, P = 0.001 for cardiovascular; HR 1.278, 95% CI 1.028–1.589, P = 0.027 for cardiac). In the Cox proportional hazards model, the difference between the *PON1* rs705379 TT and CC genotypes concerning mortality from CHD and its complications was connected only with the TT homozygosity (HR 1.549, 95% CI 1.064–2.255, P = 0.023).

There were no associations between rs662 and rs854560 and cardiovascular, cardiac, and vascular mortality in HD patients.

## Discussion

HD patients are burdened with dyslipidaemia, CHD, MI, and ICS more frequently than the general population^40–42^. This study shows several correlations between *PON1* genetic polymorphisms (missense variants rs662 and rs854560, and promoter variant rs705379) and circulating functional protein PON1 concerning atherosclerotic phenotypes. Only associations, which remained significant among crucial demographic and clinical variables, were considered as having a meaningful contribution to dyslipidaemia and atherosclerotic complications.

The involvement of *PON1* SNVs in dyslipidaemia, cardiovascular diseases, and mortality of the examined HD subjects may be summarized as follows: Low activity alleles of *PON1* rs662 (A) and rs854560 (T) contribute to the higher prevalence of atherogenic dyslipidaemia diagnosed by the TG/HDL-cholesterol ratio ≥ 3.8 but are not related to untransformed parameters of serum lipid profile and dyslipidemic patterns established by K/DOQI for kidney disease patients^39^. The *PON1* rs854560 T allele is involved in the higher prevalence of ICS. The *PON1* rs705379 genotype composed of low activity alleles (TT) contributes to cardiovascular mortality, cardiac deaths, and CHD mortality and its complications. The protein product of *PON1* is paraoxonase. Normalized serum PON1 (paraoxonase) activity positively associates with atherogenic dyslipidaemia. All mentioned above findings are consistent, although not all previously found associations (with CHD, MI) were revealed in HD patients at a statistically significant level.

Associations between *PON1* SNVs and serum lipid parameters or dyslipidaemic profiles were evaluated among possible confounding variables like age, gender, diabetic nephropathy, and lipid-modifying treatment. It was shown that simvastatin increased the *PON1* promoter's activity in a dose-dependent manner in expression cassettes transfected into HepG2 cells^29^. In HuH7 cells, statins (pravastatin, simvastatin, fluvastatin) downregulated the *PON1* promoter by 30–50% in a dose-dependent manner. In contrast, the PON-1 secreted enzymatic activity and mRNA levels were increased by the active form of fenofibrate, fenofibric acid, approximately 70%^16^. In HD patients, statin treatment was independently positively related to PON1 concentration^12^. However, in another study on HD subjects, there were no significant differences in PON1 activity that could be related to statins^43^. In our study, the results of associations between *PON1* SNVs and atherogenic dyslipidaemia seem to be not influenced by lipid-modifying therapy. Still, the correlation between rs662 and serum HDL-cholesterol became not significant in the model, including lipid-modifying treatment.

Studies on non-uremic subjects are showing higher CHD risk associated with the *PON1* rs662 A (Q192) and the *PON1* rs854560 T (M55) alleles, which correspond with the low activity PON1 isoform^44,45^. On the other hand, the *PON1* rs662 G (192R) and the *PON1* rs854560 A (L55) alleles, which are related to the high activity PON1 isoform, were also found to be associated with atherogenic serum lipid profile^46^ and common in atherosclerotic diseases (CHD, ICS) in the general population^47,48^. The *PON1* rs662 G (192R) and the *PON1* rs854560 A (L55) variants were suspected to be associated with CHD, particularly in diabetes mellitus, cigarette smoking, and older age^24,49–51^. There are also studies not indicating the association between the *PON1* rs662 and CHD^52^. In the examined HD patients, among three *PON1* SNVs (rs705379, rs854560, rs662), being in a weak LD, none showed a significant relationship with CHD or type 1 MI. In studies by Garin et al.^24^ and Imai et al.^47^ on non-uremic patients, which documented the associations of rs854560 and rs662 with CHD, respectively, criteria for the CHD diagnosis were much more spectacular than in our study: transmural MI or positive coronary angiogram^24^ or over 50% narrowing in at least one major coronary artery^47^. The analysis of HD cases with CHD diagnosed by such criteria could provide different results than currently presented.

In Polish HD patients, we revealed the association only between *PON1* rs854560 and ICS in the dominant mode of inheritance. In the Han Chinese population without evident kidney diseases, rs705379 was significantly associated with ICS, also in the dominant way of inheritance^53^.

*PON1* rs662 and *PON1* rs854560 SNVs were associated with serum PON1 activity in HD patients in the previous studies. In Portuguese (n = 183)^43^ and Hungarian (n = 20)^54^ HD patients, the highest PON1 activity was shown in the *PON1* rs662 GG homozygotes, the lowest—in the AA homozygotes. Concerning *PON1* rs854560 in HD patients, Portuguese AA homozygotes showed the highest PON1 activity, whereas TT homozygotes—the lowest^43^. Japanese HD patients^14^, like Polish HD subjects in this study, did not differ in PON1 activity concerning *PON1* rs854560 polymorphisms. To our knowledge, *PON1* − 108C>T (rs705379) polymorphism was not examined in HD patients, but in healthy Japanese subjects, there was a decrease in PON1 activity for − 108T individuals when compared to those with the − 108C polymorphism^16^. Therefore, our study did not reveal significant differences in serum PON1 activity and the PON1/HDL-cholesterol ratio concerning *PON1* SNVs previously associated with lower serum activity of anti-atherogenic PON1^1,43,54^. However, the low-expression alleles/genotypes of these SNVs corresponded with atherogenic dyslipidaemia (rs662, rs854560), ICS (rs854560), or cardiovascular mortality (rs705379). It places them among unfavourable genetic inheritance.

Serum PON1 activity in the examined HD patients (median 101.0 U/L, range 27.7–212.9 U/L) places within the broad range of values observed in HD groups by other authors (58.0 ± 36.7 U/L^8^–258.3 ± 42.51 U/L on NaCl-stimulation^55^). Gugliucci et al. showed the closest to ours (101.1 ± 41.1 U/L) in non-diabetic HD subjects^56^. PON1 activity was not associated with diabetes (diabetic nephropathy) in the examined HD patients, HD subjects studied by Varga et al.^57^, and Itahara et al.^58^. In another study, non-diabetic HD subjects showed higher PON1 activity than type 2 diabetics^59^.

Only the univariate analyses indicated inverse correlations between serum PON1 activity and mixed dyslipidaemia by K/DOQI and serum TG concentration. In HD patients, serum PON1 (concentration or activity) was already associated with serum lipids, mainly HDL-cholesterol and HDL subclasses^60,61^. HD patients showing CHD had lower PON1 activity than subjects without CHD^62^. In HD men but not in HD women, PON1 concentrations were lower in CHD subjects than in those without CHD, although serum PON1 concentration was not gender-dependent^13^. The normalized serum PON1 activity in healthy men was the highest in subjects with the lowest HDL-cholesterol values^63^. In our study, the PON1/HDL-cholesterol ratio positively correlated with the male gender. However, serum PON1 activities were similar in subjects with and without studied cardiovascular diseases, independently on gender (Supplementary Table S6). Although our results indicate that serum PON1 activity attenuates atherogenic serum lipid patterns, circulating PON1 was not associated with already developed comorbidities related to atherosclerosis (CHD, MI, ICS).

Numerous factors influencing serum PON1 activity may mask its association with the genetic background or clinical and laboratory phenotypes. Erythropoietin, widely used in HD subjects, was shown to elevate PON1 activity in predialysis patients^31^. In HD patients, zinc supplementation^32^ or decaffeinated green tea extract (catechins)^33^ increased the activity of PON. Nandrolone decanoate decreased PON activity in HD patients^34^. Acrolein, both air pollution and cigarette smoke component^36^, inactivates PON1^37^. The acrolein levels are usually low in outside air (0.20 ppb in urban air and 0.12 ppb in rural air). However, in large cities, acrolein pollution reaches 5.6 ppb.^36^. Thus, places of settlement (city, village), different in air pollution, may influence PON1. Age^13,58^, dialysis duration^30,58,64^, serum urea and creatinine^30,58^, vitamin C administration^33,65^, and body mass index^57,66^ yielded ambiguous results concerning their influence on PON1 activity. Lipid-modifying treatment^12,16,29^, advanced glycated end-products^61^, and C-reactive protein (CRP) level^66^ are also mentioned among possible modifiers of PON1 activity. An impact of secondary hyperparathyroidism on PON1 is also possible. Paraoxonases have two binding-calcium sites. One of two calcium atoms and a phosphate ion lie at the bottom of the PON1active-site cavity^67^. Calcium chelators inhibit PON1 (paraoxonase) activity. Complete removal of calcium led to irreversible inactivation of PON1 activity. Human serum PON1 requires calcium for enzymatic activity, and calcium is needed for maintaining PON structural stability^35^.

This study checked several factors mentioned above for association with serum PON1 activity and the PON1/HDL-cholesterol ratio. In HD patients, paraoxonase concentration was not associated with gender in multiple regression analysis^13^. Our study did not reveal a relationship between serum PON1 activity and gender, but such an association was demonstrated for normalized PON1 activity (the PON1/HDL-cholesterol ratio). Male gender and cigarette smoking, being traditional risk factors for CHD and MI^68^, were positively related to the higher PON1/HDL-cholesterol ratios. Unexpectedly, village settlement also appeared to be positively associated with the higher PON1/HDL-cholesterol ratio. Maybe unhealthy rural residents (HD patients) prefer living in indoor air that contains more acrolein than outdoor air (< 0.02 to 12 ppb but can be higher if residents smoke tobacco at home)^36,69^. On the other hand, positive correlations of the PON1/HDL-cholesterol ratio with village settlement as well as zinc supplementation could be related to their relatively higher positive influences, if any, on PON1 activity than on serum HDL-level. Correlations between these variables need further studies. In HD patients, the higher urine output represents better residual renal function and is generally connected with healthier cardiovascular status^70,71^. It is well established that the decreased kidney function is associated with reduced basal and stimulated PON1 activity^72^. Following this finding is the inverse association of the PON1/HDL-cholesterol ratio with urine output in the examined HD patients. Calcium-phosphorus parameters, disturbed in the examined HD patients according to serum levels of calcium, phosphorus, and parathyroid hormone, did not correlate with serum PON1 at the taken method of the significance validation.

Ikeda et al.^14^ have found that PON1 concentration (but not paraoxonase activity or *PON1* rs662 and *PON1* rs854560) was involved in cardiovascular mortality in HD patients. In the examined HD patients, the *PON1* rs705379 TT genotype was associated with cardiovascular mortality, specifically with cardiac deaths and mortality due to CHD and its complications. *PON1* rs705379 is the main contributor to serum PON1 variation, accounting for about 13% of the disparity in arylesterase activity^73^. In the study by Gupta et al.^74^, the *PON1* rs705379 CT and TT genotypes corresponded in non-diabetics with the lower PON1 activity and CHD. However, this significance did not persist in multiple regression analysis. The TT genotype was also associated with low serum PON1 activity and an increased CHD risk in type 2 diabetic patients^75^. In the examined HD patients, associations between rs705379 and CHD were not demonstrated and did not explain cardiac deaths. The TT genotype of *PON1* rs705379 independently correlated with type 2 diabetic nephropathy as a cause of end-stage renal disease^76^. This study shows that the TT rs705379 genotype and type 2 diabetic nephropathy are independent predictors of cardiovascular (and cardiac) mortality in HD patients. Maybe, attenuated antioxidant, anti-inflammatory, anti-apoptosis, anti-thrombosis, and anti-adhesion properties of PON1^2–4^ are involved in overall cardiovascular mortality, not only in deaths due to CHD and its complications.

Our study indicates that *PON1* SNVs correlate with HD patients' clinical parameters (dyslipidaemia, ICS, cardiovascular mortality). These data suggest that abnormalities resulting from long-term atherosclerotic disturbances, like ICS or cardiovascular deaths, are satisfactorily associated with *PON1* SNVs in HD patients. Associations of non-normalized serum PON1 activity with simultaneously shown clinical variables were limited to mixed dyslipidaemia by K/DOQI guidelines and serum TG levels in unadjusted analyses. Several studies^21,77–80^ have demonstrated the usefulness of the PON1 status in the evaluation of clinical PON1 associations. The PON1 status includes simultaneous determination of paraoxonase and diazoxonase activities^21,77–79^ or PON1 activity and concentration^80^. Futurę studies incorporating the PON1 status are warranted in establishing PON1 relationships in end-stage renal disease patients treated with HD.

Our study shows multifaceted associations of *PON1* with dyslipidaemia, ICS, and cardiovascular mortality in HD patients providing arguments for the consideration of *PON1* as a therapeutic target in the prevention of atherosclerosis and its complications in uremic patients. At present, by implementing therapeutic lifestyle changes (aerobic exercises) and niacin, at least in men with metabolic syndrome, we can increase PON1 activity and PON1 concentration^81^. In the future, gene therapy may be a solution retaining multifunctional *PON1* capacity.

## Conclusions

1. In HD patients, there are associations of *PON1* SNVs with the prevalence of atherogenic dyslipidaemia diagnosed by the TG/HDL-cholesterol ratio ≥ 3.8 (higher for low activity alleles of rs662 and rs854560), a frequency of ICS (higher for the rs854560 low activity allele), and cardiovascular mortality, specifically with cardiac deaths, as well as mortality due to CHD and its complications (higher in *PON1* rs705379 low activity homozygotes).
2. The normalized serum PON1 activity (the PON1/HDL-cholesterol ratio) positively associate with atherogenic dyslipidaemia, male gender, and cigarette smoking, while a negative correlation occurs with urine output.
3. Correlations between the PON1/HDL-cholesterol ratio, living in the rural area, and zinc supplementation need further studies.
4. Multifaceted associations of *PON1* with dyslipidaemia, ICS, and cardiovascular mortality provide arguments for the consideration of *PON1* as a therapeutic target in the prevention of atherosclerosis and its complications in uremic patients.

## Patients and methods

### Patients

For *PON1* SNVs genotyping, we used DNA samples of HD patients, which we collected from January 2009 to June 2019. As we planned to analyse serum lipid data, probes of patients showing secondary causes of dyslipidaemia (hypothyroidism, alcohol abuse, medication with anticonvulsants, corticosteroid therapy) and cachectic conditions causing decreases in serum lipids (neoplasms, enteropathies, liver cirrhosis), were a priori excluded. In total, we included 1407 patients. In all these subjects, we recorded demographic and clinical parameters, including dates of birth, the start of RRT, and death, gender, cause of end-stage renal disease, and cause of death, as appropriate. Concerning diabetes mellitus, we included only patients with type 2 diabetes as a cause of end-stage diabetic nephropathy. Evidence for dyslipidaemia, lipid-modifying therapy, CHD, including MI, and ICS was also gathered in all patients, if possible. To be enrolled in the dyslipidaemia study, patients had to possess serum lipid data obtained when they presented stable clinical status at least six weeks before serum lipid measurement and did not have the following exclusion criteria: blood or plasma transfusion as well as more significant surgery during three months preceding blood sampling for lipids. The demographic, clinical, and laboratory data and the mode of lipid-modifying therapy were taken at the time of blood collection for lipids. The patients’ treatment was provided by nephrologists in the dialysis centres and was not influenced by the study. CHD, MI, and ICS data we gathered using the entire available period of RRT.

Data were collected and identified by patients’ names and surnames except from results obtained in two dialysis facilities where the code system existed.

From the cohort mentioned above, we enrolled patients for testing PON1 activity. Therefore, these subjects fulfilled the enrolment criteria described for the entire HD population genotyped for *PON1* SNVs. Blood samples for serum PON1 activity were collected in two collaborating dialysis centres for adult patients (≥ 18 years). In one centre, low-flux dialyzers were used; in another one—high flux dialyzers were applied. Patients were dialyzed in May–June 2019, three times a week. They were randomly selected from currently available stable patients who gave written consent (n = 93).

In patients tested for serum PON1 activity, we additionally recorded a place of settlement (city, village), a history of parathyroidectomy, actual dry body mass, ESA administration, treatment with cinacalcet hydrochloride and phosphate binders, cigarette smoking, zinc supplementation, green tea intake, vitamin C and D supplementation, and nandrolone decanoate usage. Blood samples for serum PON1 activity were collected in these 93 patients together with routine laboratory parameters, which included serum concentrations of lipids, urea, creatinine, haemoglobin, CRP, total Ca, P, intact parathyroid hormone, and activity of liver enzymes.

All study subjects were Caucasians.

We diagnosed dyslipidaemia according to the K/DOQI guidelines, which were elaborated taking into account the specificity of lipid abnormalities in chronic kidney disease subjects, including HD individuals^39^. Patients diagnosed as dyslipidaemic by serum LDL-cholesterol ≥ 100 mg/dL, we referred to as hyper-LDL-cholesterolaemic. Those showing non-HDL-cholesterol ≥ 130 mg/dL and TG ≥ 200 mg/dL were described as hyper-TG/hyper-non-HDL-cholesterolaemic. HD subjects showing dyslipidaemia by serum LDL cholesterol ≥ 100 mg/dL and simultaneously occurring non-HDL cholesterol ≥ 130 mg/dL and TG ≥ 200 mg/dL are referred to as having mixed dyslipidaemia^82^. The remaining patients were diagnosed as non-dyslipidaemic by K/DOQI criteria. We used the atherogenic index (the TG/HDL-cholesterol ratio) to interpret the atherogenic pattern of dyslipidaemia. The ratio of ≥ 3.8 was considered as indicating atherogenic dyslipidaemia because this ratio was reliable for identifying atherogenic LDL phenotype B in men and women^38^. HD subjects with the TG/HDL-cholesterol ratio < 3.8, we described as patients without atherogenic dyslipidaemia.

CHD was diagnosed based on medical history, electrocardiograms, exercise stress tests, and, in some cases, coronary angiography or computed tomography. From CHD patients, we selected subjects who underwent MI, diagnosed using medical history, characteristic electrocardiographic abnormalities, and elevated levels of cardiac markers of cardiomyocyte damage. Patients with ST-elevation MI and non-ST-elevation MI were included in this study. Type 1 MI^83^ was recognized in all of them. Clinical data and computed tomography were used for the determination of ICS.

### Blood sampling

Blood probes for *PON1* genotyping and serum PON1 activity were taken before a midweek dialysis session when HD patients had collected blood for routine periodical laboratory testing. Monovette tubes (SARSTEDT, Nümbrecht, Germany) were used for venous blood sampling. Tubes containing the EDTA anticoagulant were applied for DNA analyses and blood morphology. If serum was needed, blood samples were drawn to Monovette tubes allowing the blood clotting (no anticoagulant). PON1 activity, cholesterols, TG, creatinine, urea, CRP, albumin, calcium, phosphorus, alkaline phosphatase (ALP), and parathyroid hormone were determined in serum.

### PON1 genotyping

DNA was extracted from blood lymphocytes by the salt-out extraction method. Coded DNA samples were stored at – 75 ° C and genotyped.

*PON1* SNVs designated as rs662 (Q192R, 575A>G), rs854560 (L55M, 163A>T), and rs705379 (− 108C>T) were selected for genotyping.

*PON1* rs662 was genotyped using a high-resolution melting curve analysis on the Light Cycler 480 system (Roche Diagnostics, Germany). Analysis of *PON1* rs854560 and rs705379 was performed using predesigned TaqMan SNV Genotyping Assay according to the manufacturer’s instructions provided by Applied Biosystems (Applied Biosystems, Foster City, CA).

### Serum PON1 activity

We determined circulating PON1 activity assessing catalytic efficiency for paraoxon hydrolysis (paraoxonase activity) with the use of a commercially available kit produced by Rel Assay Diagnostics, Mega Tıp (Gaziantep, Turkey, REF: RL0031, LOT: NN19064P), also applied by other investigators in clinical studies^84,85^. Two reagents are used in this kit: one contains a Tris buffer and Ca ion, the second—a stabile substrate solution. According to the manufacturer, the paraoxonase assay coefficient of validation (CV%) was 4.1 for high activity sera pool, 1.7—for medium activity sera pool, and 1.5—for low activity sera pool.

### Other laboratory methods

Total cholesterol, HDL-cholesterol, and TG were measured using specific enzymatic colorimetric tests, creatinine—by kinetic colorimetric method, urea—by kinetic method, CRP—by immunoturbidimetric method, albumin—by the colorimetric method with bromocresol green, calcium—by photometric method with 5-nitro-5’-methyl-BAPTA and EDTA, inorganic phosphate—by spectrophotometric analysis based on the formation of phosphomolybdate, ALP—by the spectrophotometric method with *p*-nitrophenyl phosphate. Reagents from Roche Diagnostics GmbH (Mannheim, Germany) were applied in all mentioned above determinations. Measurements were performed on Cobas Integra 400 (Roche Diagnostics Ltd, Rotkreuz, Switzerland).

Parathyroid hormone was measured using the chemiluminescent microparticle immunoassay with the Alinity i Intact PTH Reagent Kit produced BIOKIT, S.A. (Barcelona, Spain) for Abbott GmbH & Co. KG (Wiesbaden, Germany). Blood morphology parameters were determined by flow cytometry (Sysmex, Kobe, Japan).

All methods shown above were routinely used.

### Statistical analysis

To ensure adequate power (80%) for detection of associations between tested SNVs and dyslipidaemia by K/DOQI, atherogenic dyslipidaemia, CHD, MI, or ICS, an HD patients’ risk of these diseases was taken into the calculation of the desired sample size at different ORs (Supplementary Table S7). Allele frequencies for these calculations we obtained from gnomAD Exomes, European Non-Finnish.

We tested the distribution of continuous variables by the Shapiro–Wilk test. For the presentation of non-normally distributed variables, we used a median and range (minimum–maximum). Normally distributed continuous results are shown as a mean ± standard deviation. Categorical variables are presented as a percentage of the total number.

Departure from HWE was determined by Chi-squared analysis (df = 1, P ≥ 0.05 for accordance). Modes of inheritance were created concerning alleles known as associated with low PON1 activity or concentration as the risk alleles: A for rs662, T for rs854560, and T for rs705379^86^. To test *PON1* SNVs for associations with categorical variables (types of dyslipidaemia, CHD, type 1 MI, and ICS) in the additive genetic model, we used the Cochran-Armitage test for trends^87^. As this model does not indicate a type of inheritance and may appear unpowered, especially when the suitable mode is recessive^88^, we additionally applied the dominant and recessive modes.

The Kruskal–Wallis and Mann–Whitney U tests were used for the comparison of continuous variables. Dichotomous variables were compared using Fisher's exact test. OR with 95% confidence interval (CI) and P-values were computed to show significance in the odds of tested genotype occurrence in the case group to the odds of this occurrence in the control group. Genetic associations, yielding the P < 0.05 indicating the fifth class association by the Better Associations for Disease and GEnes (BADGE) system^89^, were verified in the multiple regression analysis or logistic regression, as appropriate, comprising clinical variables (age, gender, diabetic nephropathy, and lipid-modifying treatment when tested variables were associated with serum lipid profile; age, diabetic nephropathy, sex, and RRT duration if the association with ICS was determined).

The linear regression was used to determine the associations among serum PON1 activity or the PON1/HDL-cholesterol ratio and patient characteristics. The obtained results were adjusted for traits associated with PON1 activity or the PON1/HDL-cholesterol ratio at a P-value < 0.1 in unadjusted analyses. Multiple regression was used for adjustment. Due to a relatively small number of subjects tested for serum PON1 activity, we have chosen five variables (gender, cigarette smoking, urine output, living in a rural area, and serum phosphorus), which could influence a relationship between PON1 activity and the examined phenotypes. In the regression analyses, there was no normality for all included data. However, the outstanding values did not exceed the 3 sigma limit in any case, so we used this statistical approach. The results are presented as the regression coefficient (β) ± standard error (SE).

Survival analyses included patients who died between the start of their RRT and October 21, 2019. We evaluated cardiovascular mortality and separately cardiac and vascular mortalities. Among cardiac mortality, CHD deaths and CHD complications (heart failure, MI, sudden death) were analysed. We also computed deaths separately due to ICS. For survival analysis, we applied the Kaplan-Meyer method with the log-rank test. Mortality was calculated between genotypes and using modes of inheritance. In the case of significance, the Cox proportional hazards model was performed. If the latter was also significant, we applied the Cox proportional hazards model that included age, gender, and diabetic nephropathy.

The previously mentioned statistical analyses were performed using Graph-Pad InStat 3.10, 32 bit for Windows, created July 9, 2009 (GraphPad Software, Inc., San Diego, California, United States) and Statistica version 13, 2017 (TIBCO Software Inc., 3307 Hillview Avenue Palo Alto, CA 94,304 USA).

Linkage disequilibrium (LD) between tested SNVs was estimated using the Haploview 4.2 software (http://www.broad.mit.edu/mpg/haploview/).

P < 0.05 were chosen as the statistically significant to facilitate decisions which data may be relevant for further analyses. Finally, only associations that yielded P < 0.05 also among clinical variables were discussed as significant.

### Ethics approval and consent to participate

All patients or their parents, as appropriate, whose DNA samples were collected and are stored in the Department of Biochemistry and Molecular Biology, Poznan University of Medical Sciences, Poznań, Poland, gave a written consent informing that all their data and DNA samples may be anonymously used in the future studies concerning uremia without additional agreements. Informed consent we obtained from all study participants tested for serum PON1 activity. The Institutional Review Board of the Poznan University of Medical Sciences, Poland, approved the research design. The study conformed to the principles set out in the WMA Declaration of Helsinki and the Department of Health and Human Services Belmont Report.

## Supplementary Information

Supplementary Information.

## Data Availability

The datasets analysed during the current study are available from the corresponding author on reasonable request.

## Abbreviations

ALP: Alkaline phosphatase
CHD: Coronary heart disease
CI: Confidence interval
CRP: C-reactive protein
CV: Coefficient of validation
ESA: Erythropoietin-stimulating agent
HD: Haemodialysis
HDL: High-density lipoprotein
HWE: Hardy–Weinberg equilibrium
ICS: Ischemic cerebral stroke
IL: Interleukin
K/DOQI: Kidney Disease Outcomes Quality Initiative
LD: Linkage disequilibrium
LDL: Low-density lipoprotein
LF-HD: Low-flux haemodialysis
MAF: Minor (variant) allele frequency
MI: Myocardial infarction
NA: Not available in all patients
OR: Odds ratio
PON1: Paraoxonase 1
*PON1*: Paraoxonase 1 gene
RRT: Renal replacement therapy
TG: Triglycerides
