# Supplementary material for: Paraoxonase 1 concerning dyslipidaemia, cardiovascular diseases, and mortality in haemodialysis patients
Source: Sci Rep. 2021 Mar 24;11:6773. doi: 10.1038/s41598-021-86231-0 (PMC7990965; doi:10.1038/s41598-021-86231-0)
Supplement: Supplementary file 1 — Supplementary Information. [file 41598_2021_86231_MOESM1_ESM.docx]

Paraoxonase 1 concerning dyslipidaemia, cardiovascular diseases, and mortality in haemodialysis patients

Alicja E. Grzegorzewska, Paulina Adamska, Ewa Iwańczyk-Skalska, Kamila Ostromecka, Leszek Niepolski, Wojciech Marcinkowski, Adrianna Mostowska, Wojciech Warchoł, Czesław Żaba, Paweł P. Jagodziński

**SUPPLEMENTARY MATERIAL**

Supplementary Table 1. Characteristics of HD subjects concerning *PON1* SNVs

| **Variables tested in both groups** | Patients genotyped for *PON1* SNVs (n = 1407) | Patients tested for PON1 activity (n = 93) | P-value^a^ |
| --- | --- | --- | --- |
| ***PON1* rs705379 (-108C>T)** | n = 1332, 94.7% | n = 78 , 83.1 % | NA |
| CC | 381 (28.6%) | 23 (29.5%) | 0.898 |
| CT | 643 (48.3%) | 38 (48.7%) | 1.000 |
| TT | 308 (23.1%) | 17 (21.8%) | 0.890 |
| CC + CT | 951 (71.4%) | 61 (78.2%) | 0.244 |
| CT + TT | 1024 (76.9%) | 55 (70.5%) | 0.216 |
| MAF | 0.473 | 0.46 | 0.805 |
| P for HWE | 0.25 | 0.86 | NA |
| ***PON1* rs854560 (163A>T)** | n = 1365, 97.0% | n = 84, 90.3% | NA |
| AA | 583 (42.7%) | 42 (50%) | 0.212 |
| AT | 623 (45.65%) | 37 (44.0%) | 0.822 |
| TT | 159 (11.65%) | 5 (6.0%) | 0.153 |
| AA + AT | 1206 (88.35%) | 79 (94.0%) | 0.152 |
| AT + TT | 782 (57.3%) | 42 (50%) | 0.212 |
| MAF | 0.345 | 0.28 | 0.212 |
| P for HWE | 0.70 | 0.39 | NA |
| ***PON1*  rs662 (575A>G)** | n = 1335, 94.9% | n = 85, 91.4% | NA |
| AA | 730 (54.7%) | 47 (55.3%) | 1.000 |
| AG | 495 (37.1%) | 31 (36.5%) | 1.000 |
| GG | 110 (8.2%) | 7 (8.2%) | 1.000 |
| AA + AG | 1225 (91.8%) | 78 (91.8%) | 1.000 |
| AG + GG | 605 (45.3%) | 38 (44.7%) | 1.000 |
| MAF | 0.268 | 0.260 | 1.000 |
| P for HWE | 0.05 | 0.56 | NA |

a - Fisher's exact test

Abbreviations: HD - haemodialysis, NA – not applicable, PON1 - paraoxonase 1, *PON1* - paraoxonase 1 gene, SNV – single nucleotide variant

Supplementary Table 2. Linkage disequilibrium values D' and r^2^ for tested *PON1* variants estimated using the Haploview 4.2 software

|  | SNV | rs662 | rs854560 | rs705379 |  |
| --- | --- | --- | --- | --- | --- |
|  | rs662 | - | 0.878 | 0.183 |  |
|  | rs854560 | 0.150 | - | 0.574 |  |
|  | rs705379 | 0.011 | 0.197 | - |  |
|  |  |  |  |  |  |
| Numbers denote D’ and r^2^ values expressed as a percentage of maximal value (1.0). | | | | | |
| D' values are presented above diagonal.  r^2^ values are presented below diagonal. | | | |  |  |

Supplementary Table 3. The expected lowest OR for probability of obtaining significance at 80% sample power in associations analyses between *PON1* SNVs and tested phenotypes

| *PON1* SNV | CHD | | | MI | | | ICS | | |
| --- | --- | --- | --- | --- | --- | --- | --- | --- | --- |
|  | Cases, n | Controls, n | OR^a^ | Cases, n | Controls, n | OR^a^ | Cases, n | Controls, n | OR^a^ |
| rs662 | 514 | 821 | 1.6 | 281 | 1054 | 1.6 | 242 | 1025 | 1.8 |
| rs854560 | 528 | 837 | 1.6 | 291 | 1074 | 1.6 | 247 | 1047 | 1.8 |
| rs705379 | 510 | 822 | 1.6 | 278 | 1054 | 1.8 | 239 | 1026 | 1.8 |

^a^ – the lowest OR for probability of obtaining significance at 80% power sample

Abbreviations: CHD – coronary heart disease, ICS – ischemic cerebral stroke, MI – myocardial infarction, OR – odds ratio, *PON1* – paraoxonase 1 gene, SNV – single nucleotide variant

Supplementary Table 4. *PON1* polymorphic variants and dyslipidemic patterns by K/DOQI criteria in HD patients

| Genotypes | Non-dyslipidemic  (Reference)  n, % of all | Hyper-LDL-cholesterolemic  n, % of all | Odds ratio (95% CI),  P-value^a^ | Hyper-TG/hyper-non-HDL-cholesterolemic  n, % of all | Odds ratio (95% CI),  P-value^a^ | Mixed  n, % of all | Odds ratio (95% CI),  P-value^a^ |
| --- | --- | --- | --- | --- | --- | --- | --- |
| *PON1* rs662 (575A>G) | | | | | | | |
| AA vs. AG vs. GG | 327 (56.2%) vs. 206 (35.4%) vs. 49 (8.4%) | 194 (53.2%) vs. 138 (37.8%) vs. 33 (9%) | 0.399^b^ | 42 (55.3%) vs. 27 (35.5%) vs. 7 (9.2%) | 0.828^b^ | 91 (53.2%) vs. 68 (39.8%) vs. 12 (7%) | 0.779^b^ |
| AA + AG vs. GG | 533 (91.6%) vs. 49 (8.4%) | 332 (91%) vs. 33 (9%) | 0.925 (0.583, 1.468)  0.812 | 69 (90.8%) vs. 7 (9.2%) | 0.906 (0.395, 2.08)  0.827 | 159 (93%) vs. 12 (7%) | 1.218 (0.632, 2.347)  0.634 |
| AA vs. AG + GG | 327 (56.2%) vs. 255 (43.8%) | 194 (53.2%) vs. 171 (46.8%) | 0.885 (0.68, 1.151)  0.383 | 42 (55.3%) vs. 34 (44.7%) | 0.963 (0.596, 1.558)  0.903 | 91 (53.2%) vs. 80 (46.8%) | 0.887 (0.63, 1.249)  0.540 |
| *PON1* rs854560 (163A>T) | | | | | | | |
| TT vs. AT vs. AA | 61 (10.2%) vs. 274 (46%) vs. 261 (43.8%) | 48 (12.8%) vs. 178 (47.6%) vs. 148 (39.6%) | 0.118^b^ | 9 (11.8%) vs. 32 (42.1%) vs. 35 (46.1%) | 0.935^b^ | 24 (13.5%) vs. 79 (44.4%) vs. 75 (42.1%) | 0.752^b^ |
| AT + TT vs. AA | 335 (56.2%) vs. 261 (43.8%) | 226 (60.4%) vs. 148 (39.6%) | 1.19 (0.915, 1.548)  0.205 | 41 (53.9%) vs. 35 (46.1%) | 0.913 (0.565, 1.474)  0.175 | 103 (57.9%) vs. 75 (42.1%) | 1.07 (0.763, 1.501)  0.731 |
| TT vs. AA + AT | 61 (10.2%) vs. 535 (89.8%) | 48 (12.8%) vs. 326 (87.2%) | 1.291 (0.863, 1.931)  0.212 | 9 (11.8%) vs. 67 (88.2%) | 1.178 (0.56, 2.481)  0.689 | 24 (13.5%) vs. 154 (86.5%) | 1.367 (0.825, 2.265)  0.221 |
| *PON1* rs705379 (−108C>T) | | | | | | | |
| TT vs. CT vs. CC | 146 (24.9%) vs. 278 (47.4%) vs. 162 (27.6%) | 82 (22.2%) vs. 175 (47.4%) vs. 112 (30.4%) | 0.261^b^ | 12 (16.4%) vs. 43 (58.9%) vs. 18 (24.7%) | 0.536^b^ | 39 (23.4%) vs. 79 (47.3%) vs. 49 (29.3%) | 0.608^b^ |
| CT + TT vs. CC | 424 (72.4%) vs. 162 (27.6%) | 257 (69.6%) vs. 112 (30.4%) | 0.877 (0.658, 1.167)  0.379 | 55 (75.3%) vs. 18 (24.7%) | 1.167 (0.665, 2.048)  0.677 | 118 (70.7%) vs. 49 (29.3%) | 0.92 (0.63, 1.344)  0.696 |
| TT vs. CT + CC | 146 (24.9%) vs. 440 (75.1%) | 82 (22.2%) vs. 287 (77.8%) | 0.861 (0.632, 1.172)  0.351 | 12 (16.4%) vs. 61 (83.6%) | 0.593 (0.311, 1.132)  0.145 | 39 (23.4%) vs. 128 (76.6%) | 0.918 (0.613, 1.376)  0.760 |

a - Fisher's exact test

b - Cochran-Armitage test

Abbreviations: HD – haemodialysis, HDL - high density lipoprotein, K/DOQI - Kidney Disease Outcomes Quality Initiative, LDL - low density lipoprotein,

*PON1* - paraoxonase 1 gene, TG – triglycerides

Supplementary Table 5. Logistic regression analyses showing the association of tested *PON1* SNVs with atherogenic dyslipidaemia among other clinical variables

1. *PON1* rs662 AA + AG vs. GG, age, male gender, diabetic nephropathy, and lipid-modifying treatment

| Effect | **Atherogenic dyslipidaemia** Effect level = Yes | | | | |
| --- | --- | --- | --- | --- | --- |
|  | \| Level of effect \| \| --- \| | \| Odds ratio \| \| --- \| | \| Odds ratio  Upper 95.0% CL \| \| --- \| | \| Odds ratio  Lower 95.0% CL \| \| --- \| | \| P \| \| --- \| |
| \| Age \| \| --- \| |  | 1.000132 | 0.992154 | 1.008175 | 0.974228 |
| \| rs662 AA + AG vs. GG \| \| --- \| | AA + AG | 1.758812 | 1.103911 | 2.802238 | 0.017504 |
| \| Diabetic nephropathy \| \| --- \| | YES | 0.873053 | 0.665485 | 1.145364 | 0.327031 |
| \| Lipid-modifying treatment \| \| --- \| | YES | 1.478687 | 1.159926 | 1.885048 | 0.001591 |
| \| Male sex \| \| --- \| | YES | 1.059362 | 0.831106 | 1.350307 | 0.641382 |

1. *PON1* rs854560 AT + TT vs. AA, age, male gender, diabetic nephropathy, and lipid-modifying treatment

| Effect | **Atherogenic dyslipidaemia** Effect level = Yes | | | | |
| --- | --- | --- | --- | --- | --- |
|  | \| Level of effect \| \| --- \| | \| Odds ratio \| \| --- \| | \| Odds ratio  Upper 95.0% CL \| \| --- \| | \| Odds ratio  Lower 95.0% CL \| \| --- \| | \| p \| \| --- \| |
| \| Age \| \| --- \| |  | 1.000405 | 0.992552 | 1.008319 | 0.919842 |
| \| rs854560 AT + TT vs. AA \| \| --- \| | AT+TT | 1.284748 | 1.011103 | 1.632452 | 0.040334 |
| \| Diabetic nephropathy \| \| --- \| | YES | 0.902248 | 0.691496 | 1.177233 | 0.448540 |
| \| Lipid-modifying treatment \| \| --- \| | YES | 1.342079 | 1.056215 | 1.705312 | 0.016063 |
| \| Male sex \| \| --- \| | YES | 1.019842 | 0.803127 | 1.295036 | 0.871934 |

1. *PON1* rs854560 TT vs. AA + AT, age, male gender, diabetic nephropathy, and lipid-modifying treatment

| Efekt | **Atherogenic dyslipidaemia** Effect level = Yes | | | | |
| --- | --- | --- | --- | --- | --- |
|  | \| Level of effect \| \| --- \| | \| Odds ratio \| \| --- \| | \| Odds ratio  Upper 95.0% CL \| \| --- \| | \| Odds ratio  Lower 95.0% CL \| \| --- \| | \| p \| \| --- \| |
| \| Age \| \| --- \| |  | 1.000823 | 0.992960 | 1.008749 | 0.838004 |
| \| rs854560 TT vs. AA+AT \| \| --- \| | TT | 1.480117 | 1.036722 | 2.113147 | 0.030890 |
| \| Diabetic nephropathy \| \| --- \| | YES | 0.906382 | 0.694687 | 1.182588 | 0.468906 |
| \| Lipid-modifying treatment \| \| --- \| | YES | 1.347891 | 1.060561 | 1.713066 | 0.014661 |
| \| Male sex \| \| --- \| | YES | 1.031135 | 0.812265 | 1.308979 | 0.801146 |

Abbreviations: *PON1* – paraoxonase 1 gene, SNV – single nucleotide variant

Supplementary Table 6. Serum PON1 activity and the PON1/HDL ratio in haemodialysis patients stratified by gender

Serum PON1 activity

| Male CHD n = 15 | Male without CHD n = 40 | P^a^ |
| --- | --- | --- |
| 107.1 (38.5 – 212.9) | 102.8 (33.7 – 193.7) | 0.734 |

| Male MI n = 6 | Male without MI n = 49 | P^a^ |
| --- | --- | --- |
| 101 (57.7 – 113.1) | 105.9 (33.7 – 212.9) | 0.501 |

| Male MI n = 6 | Male without CHD n = 40 | P^a^ |
| --- | --- | --- |
| 101 (57.7 – 113.1) | 102.8 (33.7 – 193.7) | 0.590 |

| Male stroke n = 7 | Male without stroke n = 48 | P^a^ |
| --- | --- | --- |
| 101 (86.6 – 193.7) | 105.3 (33.7 – 212.9) | 0.246 |

| Female CHD n = 10 | Female without CHD n = 28 | P^a^ |
| --- | --- | --- |
| 91.4 (27.7 – 143.1) | 95 (45.7 – 188.1) | 0.464 |

| Female MI n = 9 | Female without MI n = 29 | P^a^ |
| --- | --- | --- |
| 114.3 (27.7 – 156.4) | 91.4 (45.7 – 188.1) | 0.293 |

| Female stroke n = 2 | Female without stroke n = 36 | P^a^ |
| --- | --- | --- |
| 74.0 (68.6 – 79.4) | 97.4 (27.7 – 188.1) | 0.344 |

the PON1/HDL ratio

| Male CHD n = 15 | Male without CHD n = 40 | P^a^ |
| --- | --- | --- |
| 2.88 (0.82 – 7.1) | 2.52 (0.57 – 4.97) | 0.518 |

| Male MI n = 6 | Male without MI n = 49 | P^a^ |
| --- | --- | --- |
| 2.62 (0.57 – 7.1) | 2.39 (1.05 – 3.06) | 0.405 |

| Male stroke n = 7 | Male without stroke n = 48 | P^a^ |
| --- | --- | --- |
| 2.58 (1.63 – 4.97) | 2.65 (0.57 – 7.1) | 0.757 |

| Female CHD n = 10 | Female without CHD n = 28 | P^a^ |
| --- | --- | --- |
| 2.03 (0.83 – 5.88) | 1.89 (0.67 – 3.22) | 0.987 |

| Female MI n = 9 | Female without MI n = 29 | P^a^ |
| --- | --- | --- |
| 1.81 (0.67 – 3.22) | 2.04 (0.83 – 5.88) | 0.919 |

| Female stroke n = 2 | Female without stroke n = 36 | P^a^ |
| --- | --- | --- |
| 1.83 (1.63 – 2.04) | 1.99 (0.67 – 5.88) | 0.822 |

a - Mann-Whitney U test

Abbreviations: CHD - coronary heart disease, HDL – high density lipoprotein, MI *–* myocardial infarction, *PON1* - paraoxonase 1 gene

Supplementary Table 7. Results of sample size calculations in haemodialysis patients concerning dyslipidaemia by K/DOQI, atherogenic dyslipidaemia, CHD, MI, and ICS

| **Allele frequency (gnomAD Exomes, European Non-Finnish):** |
| --- |
| rs662 - 0.281 |
| rs854560 - 0.368 |
| rs705379 - 0.458 |

**Dyslipidaemia by K/DOQI**

| **Haemodialysis population risk: 0.821** |
| --- |
| **Design: unmatched case-control (1:0.218)** |

|  | **SAMPLE SIZE** | | | | | |  |  |
| --- | --- | --- | --- | --- | --- | --- | --- | --- |
|  | **Dominant model** | | | **Recessive model** | | |  |  |
| **OR** | **rs662** | **rs854560** | **rs705379** | **rs662** | **rs854560** | **rs705379** |  |  |
| **Haemodialysis population** | **N^a^** | | | | | |  |  |
| **1.2** | 5309 | 5426 | 6171 | 19412 | 11953 | 8346 |  |  |
| **1.4** | 1571 | 1581 | 1773 | 6054 | 3701 | 2559 |  |  |
| **1.6** | 813 | 807 | 894 | 3276 | 1990 | 1365 |  |  |
| **1.8** | 525 | 515 | 565 | 2200 | 1330 | 906 |  |  |
| **2.0** | 382 | 371 | 402 | 1655 | 996 | 674 |  |  |
| **2.2** | 298 | 287 | 309 | 1333 | 799 | 538 |  |  |
| **2.4** | 245 | 233 | 249 | 1124 | 671 | 450 |  |  |
| **2.6** | 208 | 196 | 208 | 978 | 582 | 389 |  |  |
| **2.8** | 181 | 170 | 179 | 871 | 517 | 344 |  |  |
| **3.0** | 161 | 150 | 157 | 789 | 468 | 310 |  |  |
| ^a^ N is the number of cases required for the desired power (80%); the required number of controls is 0.218 x N. | | | | | | | | |

**Atherogenic dyslipidaemia**

| **Haemodialysis population risk: 0.52** |
| --- |
| **Design: unmatched case-control (1:0.923)** |

|  | **SAMPLE SIZE** | | | | | |  |  |
| --- | --- | --- | --- | --- | --- | --- | --- | --- |
|  | **Dominant model** | | | **Recessive model** | | |  |  |
| **OR** | **rs662** | **rs854560** | **rs705379** | **rs662** | **rs854560** | **rs705379** |  |  |
| **Haemodialysis population** | **N^a^** | | | | | |  |  |
| **1.2** | 1972 | 2051 | 2370 | 6814 | 4228 | 2982 |  |  |
| **1.4** | 581 | 603 | 697 | 2023 | 1253 | 882 |  |  |
| **1.6** | 299 | 310 | 359 | 1051 | 650 | 456 |  |  |
| **1.8** | 192 | 199 | 230 | 682 | 420 | 294 |  |  |
| **2.0** | 138 | 144 | 167 | 498 | 306 | 214 |  |  |
| **2.2** | 107 | 112 | 129 | 391 | 240 | 167 |  |  |
| **2.4** | 88 | 91 | 106 | 322 | 197 | 137 |  |  |
| **2.6** | 74 | 77 | 89 | 274 | 168 | 116 |  |  |
| **2.8** | 64 | 66 | 77 | 240 | 146 | 101 |  |  |
| **3.0** | 56 | 59 | 68 | 214 | 130 | 90 |  |  |
| ^a^ N is the number of cases required for the desired power (80%); the required number of controls is 0.923 x N. | | | | | | | | |

**CHD**

| **Haemodialysis population risk: 0.650** | | | |  |  |  |  |
| --- | --- | --- | --- | --- | --- | --- | --- |
| **Design: unmatched case-control (1:0.538)** | | | |  |  |  |  |
| **SAMPLE SIZE** | | | | | | | |
|  | **Dominant model** | | | | **Recessive model** | | |
| **OR** | **rs662** | **rs854560** | **rs705379** | | **rs662** | **rs854560** | **rs705379** |
| **Haemodialysis population** | **N^a^** | | | | | | |
| **1.2** | 2710 | 2797 | 3210 | | 9599 | 5936 | 4168 |
| **1.4** | 799 | 819 | 935 | | 2913 | 1793 | 1252 |
| **1.6** | 412 | 419 | 476 | | 1541 | 945 | 656 |
| **1.8** | 265 | 268 | 303 | | 1016 | 620 | 429 |
| **2.0** | 192 | 193 | 218 | | 752 | 458 | 315 |
| **2.2** | 149 | 150 | 168 | | 597 | 362 | 248 |
| **2.4** | 122 | 122 | 136 | | 497 | 301 | 205 |
| **2.6** | 103 | 102 | 115 | | 428 | 258 | 176 |
| **2.8** | 89 | 88 | 99 | | 378 | 227 | 154 |
| **3.0** | 79 | 78 | 87 | | 339 | 204 | 137 |
|  | | | | | | | |
| ^a^ N is the number of cases required for the desired power (80%); the required number of controls is 0.538 x N. | | | | | | | |

**MI**

| **Haemodialysis population risk: 0.140** |
| --- |
| **Design: unmatched case-control (1:6.143)** |

| **SAMPLE SIZE** | | | | | | |
| --- | --- | --- | --- | --- | --- | --- |
|  | **Dominant model** | | | **Recessive model** | | |
| **OR** | **rs662** | **rs854560** | **rs705379** | **rs662** | **rs854560** | **rs705379** |
| **Haemodialysis population** | **N^a^** | | | | | |
| **1.2** | 1099 | 1168 | 1375 | 3514 | 2205 | 1578 |
| **1.4** | 324 | 350 | 419 | 974 | 617 | 446 |
| **1.6** | 167 | 183 | 222 | 476 | 304 | 222 |
| **1.8** | 108 | 120 | 174 | 293 | 188 | 139 |
| **2.0** | 78 | 88 | 109 | 203 | 131 | 98 |
| **2.2** | 61 | 69 | 86 | 153 | 99 | 74 |
| **2.4** | 50 | 57 | 72 | 121 | 79 | 59 |
| **2.6** | 42 | 49 | 62 | 99 | 65 | 49 |
| **2.8** | 37 | 43 | 55 | 84 | 55 | 42 |
| **3.0** | 33 | 38 | 49 | 72 | 48 | 37 |
|  | | | | | | |
| ^a^ N is the number of cases required for the desired power (80%); the required number of controls is 6.143 x N. | | | | | | |
|  | | | | | | |

**ICS**

| **Haemodialysis population risk: 0.137** | | |  |  |  |  |  |
| --- | --- | --- | --- | --- | --- | --- | --- |
| **Design: unmatched case-control (1:6.299)** | | |  |  |  |  |  |
|  | **SAMPLE SIZE** | | | | | | |
|  | **Dominant model** | | | | **Recessive model** | | |
| **OR** | **rs662** | **rs854560** | | **rs705379** | **rs662** | **rs854560** | **rs705379** |
| **Haemodialysis population** | **N^a^** | | | | | | |
| **1.2** | 1095 | 1164 | | 1371 | 3499 | 2196 | 1572 |
| **1.4** | 323 | 349 | | 418 | 970 | 614 | 444 |
| **1.6** | 167 | 183 | | 221 | 474 | 302 | 221 |
| **1.8** | 107 | 119 | | 146 | 291 | 187 | 138 |
| **2.0** | 78 | 88 | | 108 | 202 | 131 | 97 |
| **2.2** | 61 | 69 | | 86 | 152 | 99 | 74 |
| **2.4** | 50 | 57 | | 72 | 120 | 78 | 59 |
| **2.6** | 42 | 49 | | 62 | 98 | 65 | 49 |
| **2.8** | 37 | 43 | | 55 | 83 | 55 | 42 |
| **3.0** | 33 | 38 | | 49 | 72 | 47 | 36 |
|  | | | | | | | |
| ^a^ N is the number of cases required for the desired power (80%); the required number of controls is 6.299 x N. | | | | | | | |

Abbreviations: CHD – coronary heart disease, ICS – ischemic cerebral stroke, MI – myocardial infarction
